# Pharmacokinetics and Pharmacodynamics: A Comprehensive Analysis of the Absorption, Distribution, Metabolism, and Excretion of Psychiatric Drugs

**DOI:** 10.3390/ph17030280

**Published:** 2024-02-22

**Authors:** Zainab Zakaraya, Mohammad Abu Assab, Lina N. Tamimi, Nida Karameh, Mohammad Hailat, Laila Al-Omari, Wael Abu Dayyih, Omar Alasasfeh, Mohammad Awad, Riad Awad

**Affiliations:** 1 Faculty of Pharmacy, Al-Ahliyya Amman University, Amman 19111, Jordan; nidakarameh@ammanu.edu.jo; 2 Faculty of Pharmacy, Zarqa University, Zarqa 13132, Jordan; mabuassab@zuj.edu.jo (M.A.A.); ltamimi@zuj.edu.jo (L.N.T.); 3 Faculty of Pharmacy, Al-Zaytoonah University of Jordan, Amman 11733, Jordan; m.hailat@zuj.edu.jo; 4 Faculty of Allied Medical Sciences, Al-Ahliyya Amman University, Amman 19111, Jordan; l.omari@ammanu.edu.jo; 5 Faculty of Pharmacy, Mutah University, Al-Karak 61710, Jordan; wabudayyih@mutah.edu.jo (W.A.D.); omarasasfeh90@mutah.edu.jo (O.A.); 6 Faculty of Pharmacy and Medical Sciences, University of Petra, Amman 11196, Jordan; mohammadrawad00@gmail.com (M.A.); rawad@uop.edu.jo (R.A.)

**Keywords:** pharmacokinetics, pharmacodynamics, absorption, distribution, metabolism, excretion, psychiatric drugs

## Abstract

The two main classifications of antidepressant medications are selective norepinephrine reuptake inhibitors (SNRIs) and selective serotonin reuptake inhibitors (SSRIs). Out of the available choices, selective serotonin reuptake inhibitors (SSRIs) have emerged as the most commonly prescribed option. The class demonstrates a greater degree of diversity in its structural characteristics in contrast to its neurochemical effects. Nevertheless, it is important to acknowledge that the chemical composition of a drug within this specific class does not carry substantial significance in the selection process. A comprehensive analysis of the pharmacodynamic and pharmacodynamic properties of antidepressant drugs proves advantageous for clinicians and managed care providers responsible for selecting preferred selective serotonin reuptake inhibitors (SSRIs) from a roster of authorized medications. The physicochemical characteristics, which possess considerable significance, are frequently disregarded except during the drug development stage. Pharmacodynamic properties refer to the physiological and biochemical effects that drugs exert on the human body. It is noteworthy that the inclusion of selective serotonin reuptake inhibitors (SSRIs) and serotonin–norepinephrine reuptake inhibitors (SNRIs) in a comprehensive depression management protocol may demonstrate enhanced effectiveness in clinical environments as opposed to controlled trials.

## 1. Introduction

**Figure 1:**
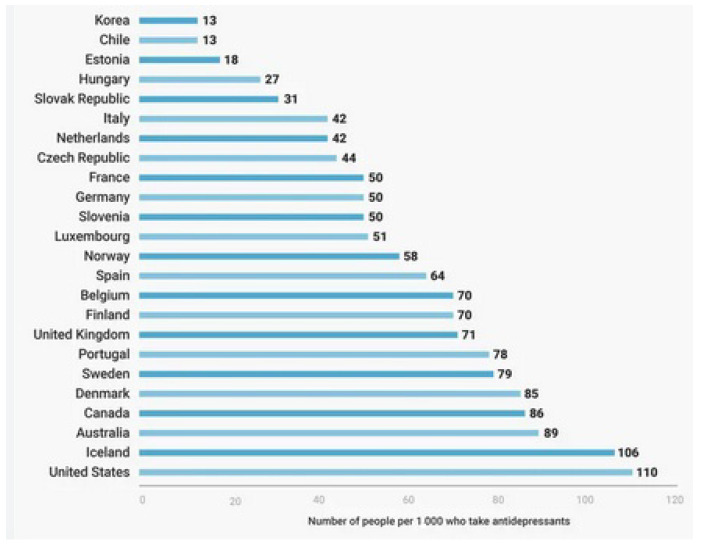
The rate of using psychiatric drugs worldwide (per 1000 persons) [4].

Psychiatric disorders are costly disorders worldwide. Their treatment and follow-up need much more time, cost, and effort [1]. The rate of these disorders has increased worldwide, with more consumption of their drugs and more exacerbated side effects. The need for novel, innovative medicines with improved pharmacological properties and fewer adverse events is mandatory [2,3]. The following Figure 1 shows the rate of the use of psychiatric drugs worldwide.

**Figure 2:**
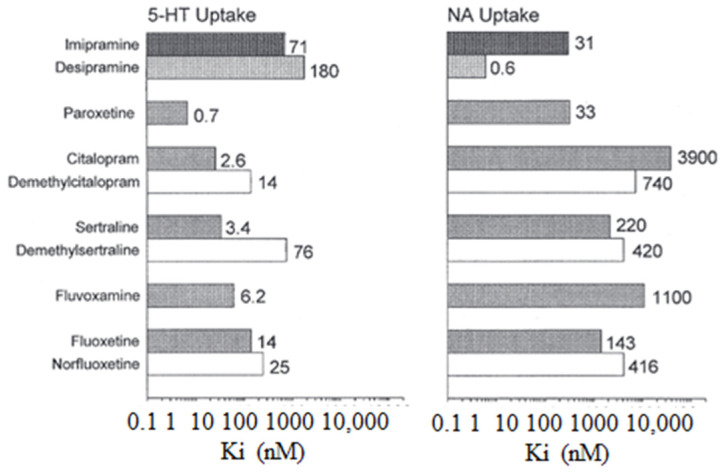
The inhibitory constants (Ki) associated with inhibiting monoamine uptake into rat brain tissue. Specifically, the focus is on the effects of imipramine, selective serotonin reuptake inhibitors, and *N*-demethylate [5].

The following Figure 2 illustrates the inhibitory constants (Ki) that inhibit monoamine uptake into rat brain tissue. Specifically, the focus is on the effects of imipramine, selective serotonin reuptake inhibitors, and *N*-demethylated metabolites, where 5-HT is also known as 5-hydroxytryptamine and NA stands for noradrenaline [4].

Selective serotonin reuptake inhibitors (SSRIs) are the first class of intentionally developed therapeutic medications in the field of psychiatry. After its introduction in Great Britain in 1983, fluvoxamine was followed by the widespread availability of fluoxetine, paroxetine, citalopram, and sertraline. Based on empirical evidence derived from clinical trials, it is widely acknowledged that selective serotonin reuptake inhibitors (SSRIs) are regarded as a viable alternative to tricyclic antidepressants (TCAs). In several countries, there has been a replacement of tricyclic antidepressants (TCAs) with alternative pharmacological treatments that are considered the initial choice for managing depression. Regarding their therapeutic efficacy, both selective serotonin reuptake inhibitors (SSRIs) and tricyclic antidepressants (TCAs) demonstrate comparable levels of potency. Selective serotonin reuptake inhibitors (SSRIs) demonstrate a noteworthy lack of potentially fatal adverse effects, such as harm to the heart and central nervous system (CNS), primarily due to their restricted receptor antagonism. Selective serotonin reuptake inhibitors (SSRIs) are widely regarded as safe and easily manageable in terms of their utilization and administration. Based on a survey conducted in Sweden, encompassing a sample size of 1202 reports documenting adverse reactions to selective serotonin reuptake inhibitors (SSRIs), the predominant occurrences reported were associated with neurological symptoms (22.4%), psychiatric symptoms (19.4%), and gastrointestinal symptoms (18%) [5]. The potential exists for a shift in the treatment approach for depression with antidepressant medications from predominantly inpatient settings to outpatient settings due to the favorable safety profile demonstrated by selective serotonin reuptake inhibitors (SSRIs).

Moreover, the application of selective serotonin reuptake inhibitors (SSRIs) has been extended beyond the management of major depression to encompass minor depression and other psychiatric disorders that have been postulated to be associated with a compromised serotonin system. The conditions above include anxiety disorders, obsessive–compulsive disorders, and premenstrual dysphoric disorders. Hence, the use of selective serotonin reuptake inhibitors (SSRIs) may be regarded as a rational and mechanism-based therapeutic strategy [6].

These abnormalities include non-linear kinetics, discrepancies between genders, variations related to age, and relevant drug–drug interactions that have clinical implications. The class of antidepressant medications known as serotonin–norepinephrine reuptake inhibitors (SNRIs) operates by impeding the reuptake of serotonin and norepinephrine neurotransmitters. These medications are frequently categorized as a group of interconnected antidepressants that function by inhibiting the process of reuptake [7]. Nevertheless, these substances commonly display discernible variations in their chemical compositions and pharmacological properties [8].

Serotonin–norepinephrine reuptake inhibitors (SNRIs) are a pharmacological class of antidepressant medications that exert their therapeutic effects by impeding the reuptake process of both serotonin and norepinephrine neurotransmitters. This article investigates the differences among serotonin–norepinephrine reuptake inhibitors (SNRIs), including factors such as the year of approval by the United States Food and Drug Administration, availability of generic versions, approved clinical indications, duration of action, metabolic processes and elimination, presence or absence of active metabolites, recommended dosing regimens, relative effects on serotonin and norepinephrine, and the temporal pattern of serotonin and norepinephrine reuptake (sequential or simultaneous). It is important to reiterate that serotonin–norepinephrine reuptake inhibitors (SNRIs) are classified as a type of antidepressant medication. Nevertheless, it is crucial to acknowledge that SNRIs exhibit a significant array of variations, which may have potential implications for the clinical treatment of patients.

**Figure 3:**
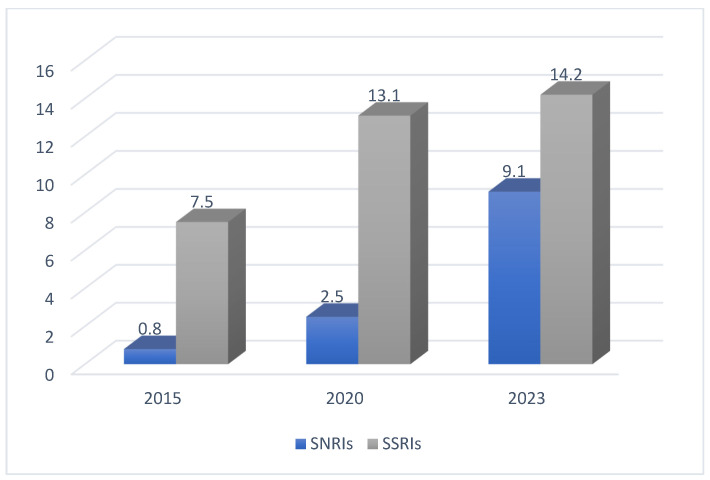
The adherence to SSRIs and SNRIs in three different years [2].

The following Figure 3 shows a comparison between SNRI and SSRI adherence in the years 2015, 2020, and 2023 [2].

## 2. Selective Serotonin Reuptake Inhibitors

### 2.1. Pharmacodynamics

#### 2.1.1. Fluoxetine

Fluoxetine was the first selective serotonin reuptake inhibitor (SSRI) to be widely introduced for clinical use in most countries. The compound being discussed is a racemic mixture comprising two enantiomers. It is essential to acknowledge that the *S*-enantiomer demonstrates a greater potency, by approximately 1.5 times, in inhibiting serotonin reuptake than the *R*-enantiomer [7].

The pharmacological differentiation between enantiomers is notably apparent in the context of the active metabolite norfluoxetine, wherein the *S*-enantiomer demonstrates approximately 20-fold higher efficacy in inhibiting reuptake than the *R*-enantiomer. In a state of equilibrium [9], it is commonly observed that the concentration of racemic norfluoxetine exceeds that of racemic fluoxetine. The levels of the *N*-demethylated metabolite are relatively higher in blood samples of *S*-norfluoxetine compared with *R*-norfluoxetine [10]. Regarding protein binding, fluoxetine exhibits a notable degree of lipophilicity and demonstrates a high affinity for plasma proteins, facilitating its distribution to the central nervous system with its active metabolite, norfluoxetine. The variability in the volume of distribution of fluoxetine and its metabolite ranges from 20 to 42 L/kg. The plasma protein binding of fluoxetine is estimated to be around 94% [7,11,12].

#### 2.1.2. Paroxetine

Paroxetine is widely regarded as the most effective serotonin reuptake inhibitor currently available for clinical application. Nevertheless, it demonstrates a relatively diminished degree of selectivity toward the serotonin reuptake site compared with fluvoxamine or sertraline. Moreover, it has been noted that it shows a similar degree of blockade of muscarinic acetylcholine receptors as the tricyclic antidepressants (TCAs) imipramine or doxepin. It exhibits even higher effectiveness in this aspect when compared with desipramine or maprotiline. Notwithstanding this attribute, the manifestation of anticholinergic adverse effects is anticipated to be confined to toxic concentrations of paroxetine, which surpass the dosages required for therapeutic efficacy [13].

Regarding protein binding, paroxetine exhibits a high degree of plasma protein binding, with around 95% of the drug being bound [14]. Paroxetine metabolism occurs mainly in the liver and is predominantly facilitated by the cytochrome enzyme CYP2D6, with additional involvement from CYP3A4 and potentially other cytochrome enzymes. The pharmacokinetics of this medicine may be affected by genetic variations in the CYP2D6 enzyme [15,16,17].

### 2.2. Pharmacokinetics

#### 2.2.1. Absorption

##### Fluoxetine

Fluoxetine is absorbed extensively after oral administration. Hepatic first-pass metabolism leads to a decrease in oral bioavailability to below 90%. Similar to other lipophilic drugs, fluoxetine has a significant volume of distribution (V_d_) ranging from 14 to 100 L/kg, indicating substantial accumulation in tissues. Fluoxetine exhibits the highest volume of distribution (V_d_) when compared with other selective serotonin reuptake inhibitors (SSRIs) [18,19]. The chemical is most highly concentrated in the lungs due to its significant abundance of lysosomes. A commonly held hypothesis suggests that the occurrence of lysosomal entrapment influences the increase in the volume of distribution (V_d_) associated with fluoxetine. Although the volume of distribution (V_d_) is larger, similar to tricyclic antidepressants (TCAs), the accumulation of this selective serotonin reuptake inhibitor (SSRI) in the brain is significantly lower compared with other SSRIs [20]. This phenomenon has been confirmed through laboratory experiments using brain tissue samples and real-time observations made on patients using fluorine-19 nuclear magnetic resonance spectroscopy (NMRS). The ratio of fluoxetine concentration in the brain to the concentration in the rest of the body is 2.6:1 in patients, whereas for fluvoxamine, the ratio is 24:1 [21].

##### Paroxetine

Paroxetine is an example of a selective serotonin reuptake inhibitor (SSRI) that demonstrates chirality and is commercially accessible as a solitary enantiomer [22]. The observed phenomenon leads to a higher level of consistency in pharmacokinetics when comparing enantiopure selective serotonin reuptake inhibitors (SSRIs), such as fluoxetine or citalopram, with racemic SSRIs. Paroxetine exhibits effective absorption from the gastrointestinal tract, albeit experiencing rapid metabolism during its initial passage through the liver [23]. A considerable portion of paroxetine, estimated to be around 36%, undergoes elimination via fecal excretion. However, less than 1% of this quantity is excreted in its unchanged state. The observed range of the volume of distribution (V_d_), which spans from 2 to 12 L/kg, demonstrates a resemblance to that of fluvoxamine [24]. The duration and dosage of administration contribute to the variability in the elimination half-life (t_1/2_). After 15 days of oral administration at a dosage of 20 mg per day, the substance exhibits an increase in its half-life (t_1/2_) by approximately 12%, leading to a range of 16.4 to 18.3 h. Furthermore, upon increasing the oral dosage to 30 mg of paroxetine per day, there is an observed increase in the half-life by more than 100%, falling within a range of 9.8 to 21.0 h. The manifestation of temporal effects becomes increasingly apparent when contrasting the integral of the curve (AUC) after a solitary administration with that after multiple administrations [25]. Even when administered at a lower dosage of 20 mg per day, a notable elevation in the area under the concentration–time curve (AUC) was observed, with values rising from 191 ng/hr/mL to 1481 ng/hr/mL [25,26]. Based on the existing body of literature, it has been observed that the bioavailability of the substance is less than 50% when administered as a single dose. However, there is a notable increase in bioavailability when multiple doses are administered [27]. Paroxetine is efficiently absorbed through the gastrointestinal system and undergoes a first metabolism in the liver. The primary drug undergoes conversion into metabolites that lack pharmacological activity. Cytochrome P450 2D6 (CYP2D6) is the primary enzyme that converts paroxetine into the paroxetine catechol intermediate. The elimination of paroxetine is characterized by a high-affinity saturable process closely associated with CYP2D6 activity and an extra low-affinity linear process [15,28]. In vitro tests suggest that CYP1A2, CYP3A4/5, and CYP2C19 have a limited role in the production of paroxetine catechol, where the results categorize the level of participation in the formation as follows: CYP2D6 has the highest rank, followed by CYP3A4, CYP1A2, CYP2C19, and CYP3A5. A simulation conducted on a population determined that CYP3A4 and CYP1A2 are the most probable enzymes involved in the metabolism of paroxetine in persons with decreased CYP2D6 activity, specifically those who are classified as CYP2D6 poor metabolizers (PMs) [29,30]. Paroxetine catechol is excreted in the form of conjugates of subsequent metabolites M-I (BRL 36610; (3S,4R)-4-(4-fluorophenyl)-3-(4-hydroxy-3-methoxyphenoxymethyl)piperidine), M-II (BRL 36583; (3S,4R)-4-(4-fluorophenyl)-3-(3-hydroxy-4-methoxyphenoxymethyl)piperidine), M-III (BRL 35961; (3S,4R)-4-(4-fluorophenyl)-3-(hydroxymethyl)piperidine), and other metabolites that are soluble in water. Catechol-O-methyltransferase (COMT) methylates it, resulting in the formation of metabolites M-I and M-II (Figure 1) [Articles: 10755376, 1531950]. We know that the enzymes responsible for forming glucuronide and sulfate conjugates have not been documented [19,31]. 

Paroxetine is a potent inhibitor of the enzyme CYP2D6, which can affect its breakdown in the body and mimic the genetic characteristics of individuals who metabolize drugs in a certain way. This can also impact the metabolism of other medications dependent on CYP2D6. Research indicates that metoprolol, clozapine, desipramine, imipramine, and a combination of dextromethorphan and quinidine can affect plasma concentrations and metabolism [16].

#### 2.2.2. Distribution

##### Fluoxetine

Fluoxetine demonstrates an extended half-life (t_1/2_) that spans a duration of 1 to 4 days. The variability in the half-life of norfluoxetine is observed with a range that encompasses a duration of 7 to 15 days [31]. Steady-state conditions require a substantial period ranging from 1 to 22 months, primarily due to the prolonged half-life. The pharmacokinetics of fluoxetine exhibit non-linear characteristics, as indicated by an amplified increase in its plasma levels following an escalation in dosage [32]. Upon administration of multiple doses, it has been observed that there is an increase in the half-life (t_1/2_) and a decrease in oral clearance compared with the administration of single doses. In the context of rats, it has been observed that the bioavailability of fluoxetine exhibits an upward trend as the dosage is escalated. This observation implies the existence of a saturable first-pass metabolism mechanism for fluoxetine in rats. Insufficient evidence exists regarding aberrations in the excretion kinetics of fluoxetine in individuals with renal dysfunction. Nevertheless, there is evidence to suggest that liver cirrhosis has a substantial impact on the plasma clearance of fluoxetine [32].

##### Paroxetine

The distribution as pharmacokinetics of paroxetine demonstrates nonlinearity, which can be delineated by two distinct mechanisms: a low-capacity, high-affinity process and a high-capacity, low-affinity process that adheres to a linear pattern. Nevertheless, this assertion is only relevant to individuals categorized as extensive metabolizers (EMs) of CYP2D6 [31]. Elderly individuals commonly demonstrate extended plasma concentrations at steady-state and the elimination half-life. Renal impairment has minimal impact on the pharmacokinetics of paroxetine, whereas hepatic dysfunction may lead to a reduction in paroxetine clearance [33].

#### 2.2.3. Metabolism

##### Fluoxetine

Fluoxetine undergoes significant metabolic conversion, leading to the generation of the active metabolite norfluoxetine alongside several other metabolites [34]. After being administered orally, the primary way fluoxetine is eliminated from the body is through excretion in the urine. A small fraction, comprising less than 10% of the total, is eliminated from the body either unchanged or as fluoxetine *N*-glucuronide. Thus far, a restricted quantity of investigations has been undertaken to scrutinize the precise CYP isoenzymes accountable for the metabolic pathways of fluoxetine. Nevertheless, the results obtained from these studies have failed to yield conclusive findings [32]. The central focus of the investigation has primarily centered on the mechanism of *N*-demethylation of fluoxetine [33,35]. According to a prior study, there was notable participation of the enzyme CYP2D6 in the *N*-demethylation process of fluoxetine among both healthy individuals and psychiatric patients who underwent a medication transition from fluoxetine to paroxetine [36]. On the other hand, the pharmacokinetics of fluoxetine and norfluoxetine are not influenced by paroxetine, a potent inhibitor of CYP2D6. There is a dearth of information about the precise enzymes that account for more than 70% of the biotransformation mechanism of fluoxetine [34,37].

Fluoxetine undergoes significant hepatic metabolism. The sole active metabolite found in norfluoxetine is produced through the demethylation of fluoxetine. Fluoxetine consists of a racemic blend of two enantiomers. *S*-fluoxetine has a somewhat more remarkable ability to block serotonin reabsorption than *R*-fluoxetine. The disparity is far more evident for the active metabolite. The reuptake-inhibiting potency of *S*-norfluoxetine is approximately 20 times greater than that of *R*-norfluoxetine. Additionally, these four chemicals also exhibit variations in their kinetics. After several weeks of treatment, the plasma concentration of both *S*-enantiomers is approximately twice as high as that of the *R*-enantiomers [10,33].

The principal elimination pathway mainly involves oxidative metabolism and conjugation. However, the identity of over half of the resulting metabolic byproducts remains unidentified. The primary route of elimination for fluoxetine is urinary excretion, with less than 10% being eliminated in its original form or as fluoxetine glucuronide. Multiple in vitro and in vivo investigations provide evidence suggesting that CYP2D6, CYP2C19, CYP2C9, CYP3A4, and CYP3A5 are involved, to some extent, in the conversion of *R*- and *S*-fluoxetine into its *N*-desmethyl metabolites [35]. The cytochrome P450 isoforms display genetic variations that impact their ability to catalyze reactions. Studies on patients with various CYP2D6 and CYP2C9 genotypes revealed that CYP2C9 primarily facilitates the process of *R*-fluoxetine demethylation, while *S*-norfluoxetine production relies heavily on CYP2D6. Simultaneously, the enantiomers of fluoxetine and norfluoxetine inhibit CYP2D6-mediated processes. Hence, the significance of CYP2C19, CYP2C9, CYP3A4, and CYP3A5 in the metabolism of fluoxetine becomes more prominent after long-term administration since the involvement of CYP2D6 is reduced due to the inhibitory effects of fluoxetine and norfluoxetine [36].

Furthermore, fluoxetine has shown inhibitory efficacy against CYP2C19, CYP2C9, and CYP3A4 in laboratory investigations. Each of these CYP isoenzymes has a role in the breakdown of many medicines. As a result, fluoxetine and norfluoxetine can potentially affect the breakdown and movement of pharmaceuticals taken together. The inhibitory effect of fluoxetine and norfluoxetine on the isoenzyme CYP2D6 has been found to cause clinically significant medication interactions with tricyclic antidepressants and neuroleptics [34].

According to the results obtained from an in vitro investigation, it was observed that the enzyme CYP2C9 plays a substantial role in the process of *N*-demethylation of fluoxetine. Furthermore, it is worth noting that the process under consideration may involve the participation of the CYP2C19 and a CYP3A isoform [38]. Nevertheless, it was found that the contribution of CYP2D6 was deemed to be inconsequential. Recent research has revealed a noteworthy association between the enzymatic activity of CYP2D6 and the elimination rate of *R*- and *S*-fluoxetine, along with *S*-norfluoxetine. Nevertheless, a lack of correlation was found for *R*-norfluoxetine [39].

**Figure 4:**
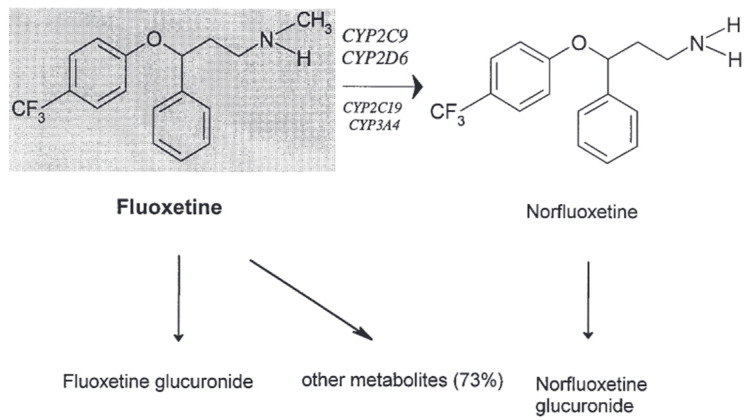
The in vivo metabolism of fluoxetine [8].

The following Figure 4 shows the metabolism of fluoxetine [8].

##### Paroxetine

Similar to other lipophilic psychotropic drugs, paroxetine undergoes extensive hepatic metabolism to generate more hydrophilic metabolites that can be eliminated from the body [14]. Metabolism involves the enzymatic breakdown of the methylenedioxy bridge through oxidation, producing a labile catechol intermediate. The intermediate undergoes subsequent methylation, forming either the meta-methoxy derivative when methylated in the meta-position or the para-methoxy derivative when methylated in the para-position. Both metabolites undergo further conjugation with either sulfuric acid or glucuronic acid [40]. There is no presumption regarding the potential contribution of any metabolites to the pharmacological effects of paroxetine [41].

**Figure 5:**
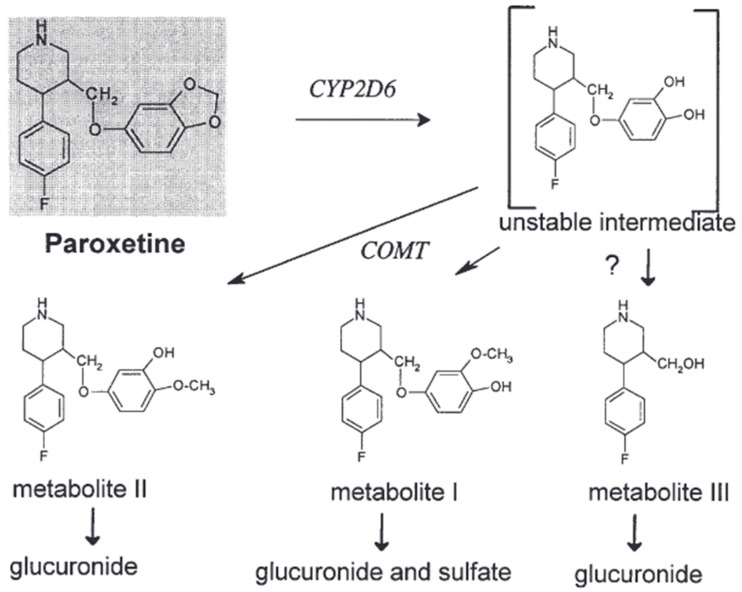
The in vivo metabolism of paroxetine [44].

The facilitation of the oxidative cleavage process is likely attributed to the presence of CYP isoenzymes, while additional enzymes are necessary for methylations. The enzymatic activity responsible for the O-methylation process is believed to be facilitated by catechol-O-methyltransferase, an enzyme involved in deactivating catecholamines and catechol estrogens. It is essential to highlight that the urine of individuals classified as poor metabolizers (PMs) exhibited notably reduced levels of the meta-O-methyl metabolite or its glucuronide and sulfate conjugates [40]. Nevertheless, the quantities of the glucuronic acid conjugate of the para-O-methyl metabolite were discovered to be comparable in both extensive metabolizers (EMs) and poor metabolizers (PMs). However, it has been observed that PMs exhibit the capacity to produce the meta-O-methyl metabolite [42]. The differences observed between EMs and PMs are more likely to be attributed to variations in their respective abilities to make the catechol intermediate rather than differences in their methylation activities [43,44] As illustrated in Figure 5 [44].

#### 2.2.4. Excretion

##### Fluoxetine

This drug exhibits renal excretion of approximately 2.5% in its unchanged form, while about 10% is excreted as norfluoxetine.

##### Paroxetine

During a 10-day post-dosing period, it was observed that approximately 64% of a 30 mg oral solution dose of paroxetine was eliminated through urinary excretion. Of this amount, 2% was identified as the parent compound, while 62% was identified as metabolites [44].

**Figure 6:**
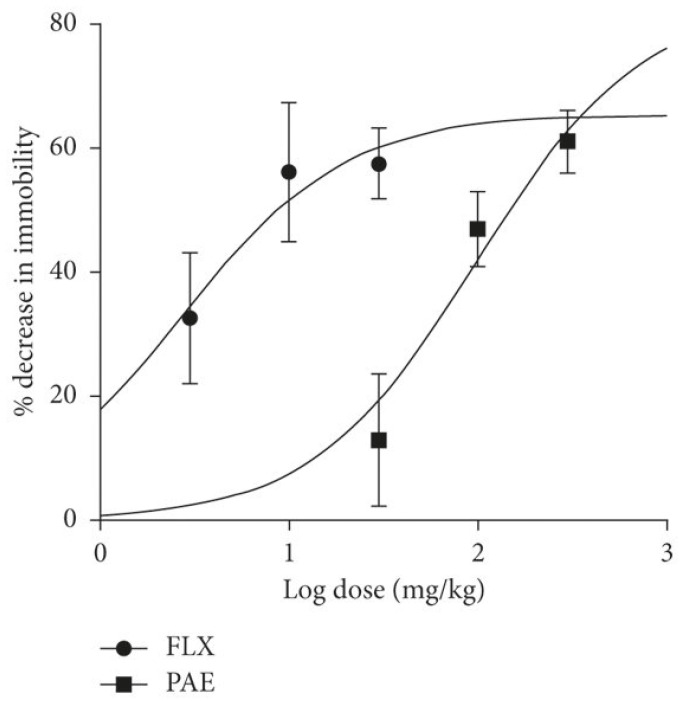
The dose–response curve that compares fluoxetine (FLX) and paroxetine (PAE) [45].

The following Figure 6 summarizes the dose–response curve comparing paroxetine and fluoxetine.

## 3. Drug–Drug Interactions between SSRIs, SNRIs, and Other Drugs

Paroxetine has been recognized as the most efficacious inhibitor of the cytochrome P450 2D6 enzyme (CYP2D6) among all selective serotonin reuptake inhibitors (SSRIs). The average inhibition constant (Ki) value for inhibiting the CYP2D6 enzyme is typically observed to be in the nanomolar range, specifically around 150 nM. This is analogous to the efficacy of quinidine (Ki = 30 nM), which is presently acknowledged as the most potent antagonist of CYP2D6 [45].

Most studies or case reports that have examined inhibitory potency have focused on evaluating the inhibition of metabolism in tricyclic antidepressants (TCAs), particularly imipramine, desipramine, or trimipramine. The potency of inhibiting *N*-demethylated metabolites of tricyclic antidepressants (TCAs), such as desipramine, is considerably more significant when compared with tertiary amines [46]. This observation is consistent with the finding that CYP2D6 plays a substantial role in eliminating secondary amines, but its importance is reduced in the metabolic elimination of tertiary amines. The extent of CYP2D6 inhibition is correlated with the concentrations of paroxetine in the circulatory system [47]. This may offer a potential rationale for the disparate findings observed in two studies investigating the effects of paroxetine on the pharmacokinetics of clozapine, an atypical antipsychotic medication. The administration of dosages surpassing 20 mg per day, with an average of 31 mg per day, led to a notable increase in clozapine plasma concentrations. In contrast, a steady daily dosage of 20 mg of paroxetine does not significantly alter clozapine concentrations [10].

Studies conducted on sertraline have indicated limited clinical implications about pharmacokinetic interactions with other medications. Nevertheless, it is essential to acknowledge that the concurrent use of sertraline and warfarin has been linked to a substantial 8.9% elevation in prothrombin time, a factor that could potentially have significant consequences [48]. Hiemke and Härtter (2000) conducted a study to investigate the potential inhibitory effects of sertraline and its *N*-demethylated metabolite, 20 C, on the enzyme CYP2D6. This investigation was carried out using in vitro experiments [11]. The results indicated a notable level of inhibitory effectiveness, as evidenced by a Ki value of 0.7 mM. However, patient research has not yielded any substantial clinical significance. There was no significant inhibitory effect observed for sertraline, even when it was administered at high chronic doses alongside desipramine. Individuals with elevated levels of CYP2D6 activity at baseline demonstrated a moderate inhibitory characteristic [10].

Sertraline has been recognized as a substrate of the cytochrome P450 3A4 (CYP3A4) enzyme, suggesting the potential for drug interactions involving this specific isoenzyme. There is no documented evidence of any observed influence on the pharmacokinetics of carbamazepine or midazolam, both of which are substrates of the enzyme CYP3A4, based on in vitro studies. A restricted yet significant body of evidence derived from two recent case reports suggests substantial hindrance in the metabolic process of clozapine due to the administration of sertraline [38].

The serum concentration of clozapine, administered at a dosage of 600 mg, and sertraline, administered at a dosage of 300 mg, was determined to be 1300 ng/mL. After the cessation of sertraline, there was a reduction of 40% in the serum concentration of clozapine [49]. Furthermore, it was observed that the administration of 50 mg of sertraline resulted in a 2.1-fold increase in the concentration of clozapine in the bloodstream of a patient who had been given 175 mg of clozapine. The observed augmentation in cognitive focus ceased after selective serotonin reuptake inhibitor (SSRI) cessation. The case reports above offer substantiating evidence for inhibiting CYP3A4 in vivo. 

The potential role of CYP2C19 and CYP2D6 in the metabolism of citalopram implies that changes in the functioning of these particular enzymes may have consequences. After an extended period of citalopram administration, there is a slight reduction in the activity of CYP2D6, which can be attributed to the inhibitory properties of *N*-desmethyl citalopram [50]. There is a dearth of evidence suggesting any significant influence of citalopram on the pharmacokinetics of CYP2C19 substrates. On the other hand, when phenothiazine neuroleptics, specifically levomepromazine, are administered concurrently, there is a notable 30% rise in the steady-state trough concentrations of citalopram. However, this increase does not yield significant clinical consequences [50]. 

Levomepromazine, a well-established inhibitor of the enzyme CYP2D6, demonstrated a significant impact by substantially increasing the steady-state concentrations of desmethyl citalopram [51]. The prolonged administration of cimetidine at high doses (800 mg/day) led to a significant decrease of 29% in the oral clearance of citalopram. This decrease was accompanied by a corresponding increase of 43% in the blood concentration of citalopram [52].

The following Table 1 shows the potential inhibitory and stimulatory effects of psychiatric drugs.

## 4. Selective Norepinephrine Reuptake Inhibitor: Duloxetine

### 4.1. Pharmacodynamics

#### Duloxetine

Duloxetine, commercially known as Cymbalta™, obtained regulatory approval from the Food and Drug Administration (FDA) in 2004 [53], thereby becoming the second selective serotonin–norepinephrine reuptake inhibitor (SNRI) to be sanctioned for utilization within the United States [19]. The therapeutic effects of serotonin–norepinephrine reuptake inhibitors (SNRIs) in the management of depression are attributed to their ability to initially impede the activity of presynaptic transporter proteins responsible for the reabsorption of serotonin and norepinephrine [54]. This process inhibits the reuptake of these neurotransmitters, causing changes in various homeostatic mechanisms, ultimately leading to increased activation of postsynaptic receptors. However, there is variability in the binding affinity of serotonin–norepinephrine reuptake inhibitors (SNRIs) toward the serotonin and norepinephrine transporter. Desvenlafaxine, duloxetine, and venlafaxine demonstrate higher efficacy in the inhibition of serotonin reuptake relative to norepinephrine reuptake. In contrast, levomilnacipran and milnacipran exhibit a predilection for inhibiting norepinephrine reuptake [35].

**Figure 7:**
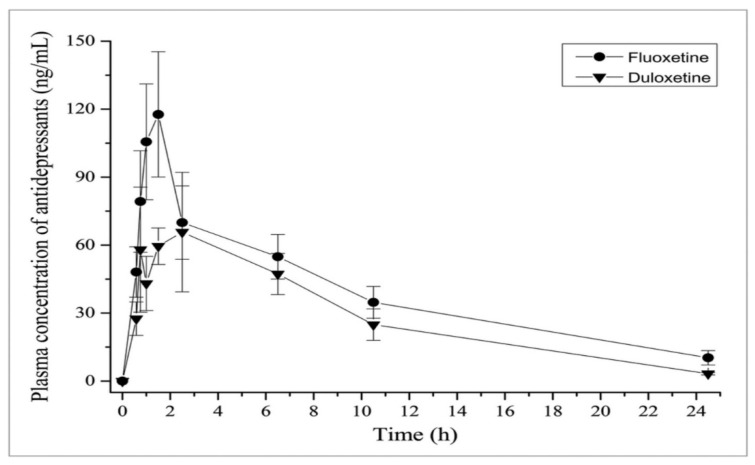
Inhibitory effects as a comparison between fluoxetine and duloxetine regarding pharmacokinetics [16].

The following Figure 7 compares the inhibitory effects of both duloxetine and fluoxetine [16].

Serotonin–norepinephrine reuptake inhibitors (SNRIs) are frequently characterized as dual-action agents [55]. However, the degree to which serotonin and norepinephrine reuptake is inhibited depends on the dosage. For example, it is evident that venlafaxine primarily acts as a selective serotonin reuptake inhibitor (SSRI) when given a daily dose of 75 mg [56]. At higher dosages, specifically at 225 mg/day and 375 mg/day, venlafaxine significantly affects the norepinephrine transporter [9]. In contrast, lower doses of levomilnacipran demonstrate greater efficacy in inhibiting the reuptake of norepinephrine relative to serotonin, displaying an approximate twofold discrepancy [57]. However, it has been observed that doses of levomilnacipran equal to or exceeding 40 mg per day demonstrate a significant inhibitory effect on 90 percent of the norepinephrine reuptake and 80 percent of the serotonin reuptake. All the estimates above were obtained with indirect measures and the calculation of group means [58].

Pharmaceutical agents that hinder the reuptake of both serotonin and norepinephrine have exhibited marginally superior effectiveness in the management of unipolar major depression when compared with selective serotonin reuptake inhibitors (SSRIs). A thorough examination was performed on 93 randomized trials, encompassing a patient population exceeding 17,000 individuals [7]. This analysis aimed to assess and compare the effectiveness of dual-action agents compared with selective serotonin reuptake inhibitors (SSRIs) for treating patients. The findings of the study revealed that a more significant percentage of patients administered dual-action drugs (64 percent) exhibited a favorable response in comparison with those who received selective serotonin reuptake inhibitors (SSRIs) (59 percent). Notably, while this disparity showed statistical significance, the magnitude of the effect was relatively modest [39].

### 4.2. Pharmacokinetics

#### 4.2.1. Absorption

##### Duloxetine

Duloxetine was first introduced as a therapeutic option for the management of diabetic peripheral neuropathy, establishing itself as the first medication to be approved for this specific condition in the United States [59]. Duloxetine has received approval from the Food and Drug Administration (FDA) for the treatment of major depression, generalized anxiety disorder, musculoskeletal pain, fibromyalgia, and osteoarthritis since its establishment. As a result, duloxetine exhibits the most extensive range of approved indications by the Food and Drug Administration (FDA) compared with other serotonin–norepinephrine reuptake inhibitors (SNRIs) [10]. Duloxetine, in comparison with venlafaxine, has been found to possess a range of clinical indications for nonpsychiatric conditions, with each indication corresponding to a specific type of pain syndrome. Duloxetine and venlafaxine demonstrate structural dissimilarity, as duloxetine is characterized by a chemical structure consisting of three distinct rings, two of which are near each other. Duloxetine was identified as a prospective candidate for generic formulation in late 2013 [12].

**Figure 8:**
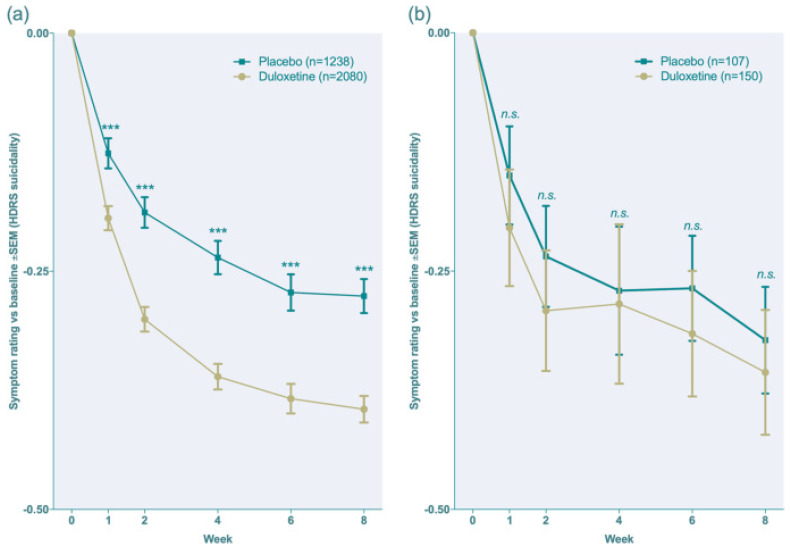
The efficacy of duloxetine when compared to placebo (as a clinical response) [12], (**a**,**b**). *** *p* < 0.001, ns = no significance.

The following Figure 8 illustrates the drug response when compared to placebo to show the efficacy of duloxetine [12].

#### 4.2.2. Distribution

##### Duloxetine

The duration of duloxetine’s half-life is approximately 12 h. Even though duloxetine undergoes metabolism, the resulting metabolites are either short-lived or do not possess significant biological effects. In other words, the metabolites of duloxetine have minimal or no substantial clinical activity [60]. The numerical values of 6 and 7 were presented [61].

Duloxetine undergoes primarily hepatic metabolism via the P-450 isoenzyme system, specifically the 2D6 and 1A2 isoenzymes. This suggests the possibility of drug interactions and a vulnerability to genetic polymorphism, particularly for the 2D6 isoenzyme. The recommended dosage is administered once daily [13].

#### 4.2.3. Metabolism

##### Duloxetine

Duloxetine primarily undergoes hepatic metabolism through two cytochrome P450 isozymes, namely, CYP2D6 and CYP1A2. Pharmacologically inactive metabolites are present in the circulation. Duloxetine exhibits moderate inhibition of the cytochrome P450 2D6 enzyme.

#### 4.2.4. Excretion

##### Duloxetine

**Figure 9:**
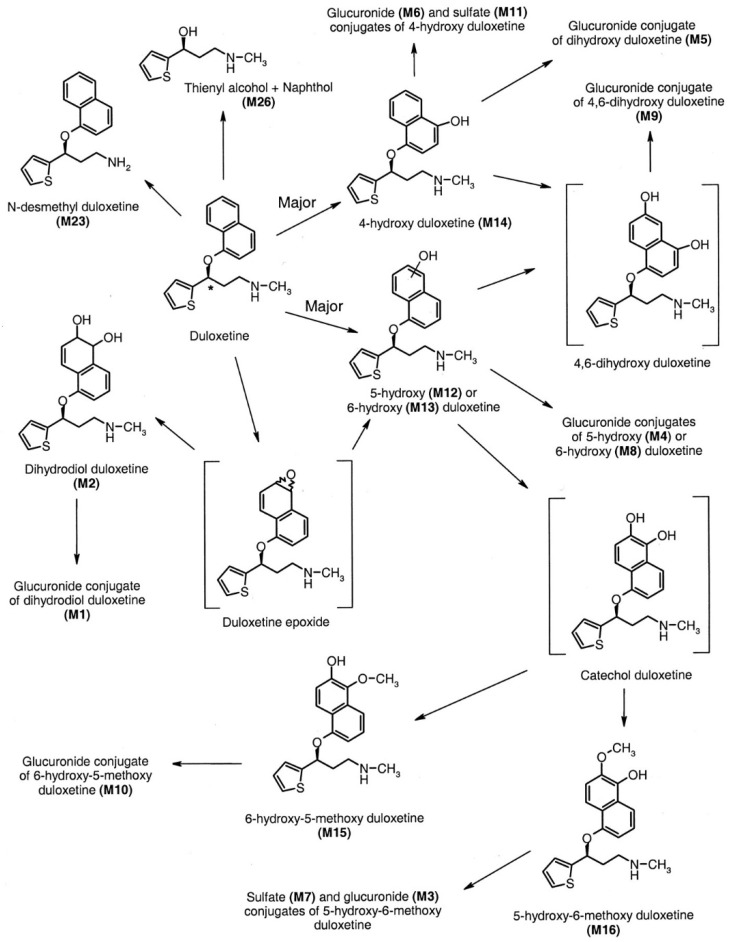
The in vivo metabolism of duloxetine [12].

It has been observed that food consumption has a moderating impact on the absorption rate without influencing the extent of absorption. The concurrent administration of medications with food has been observed to potentially mitigate the incidence of nausea, a frequently reported adverse effect of serotonin–norepinephrine reuptake inhibitors (SNRIs). Duloxetine demonstrates resemblances to both selective serotonin reuptake inhibitors (SSRIs) and tricyclic antidepressants (TCAs) about its substantial protein binding capacity, predominant hepatic clearance, and negligible renal excretion, accounting for less than 1 percent of the drug in its unaltered state. In contrast, the remaining serotonin–norepinephrine reuptake inhibitors (SNRIs) demonstrate comparatively reduced levels of protein binding [16]. Moreover, the clearance process of these drugs is significantly influenced by their elimination via renal excretion [35]. Furthermore, a higher percentage of the medication is eliminated unchanged via the urinary system. As a result, renal disease is more prone to require dosage modification in administering these serotonin–norepinephrine reuptake inhibitors (SNRIs) [4,19] As shown in Figure 9 [12].

## 5. Conclusions

After careful consideration of the past ten years of widespread use of selective serotonin reuptake inhibitors (SSRIs), it is clear that the introduction of SSRIs has not only brought about a new class of medications but has also shifted our attention toward the importance of pharmacokinetic properties in the overall functioning of drugs. The essential attributes of a chemical substance should not be conflated with the pharmacokinetic properties of a pharmaceutical compound. Differences in traits can manifest in both inter-individual and intra-individual contexts. To provide safe and effective care to patients, clinicians must possess a comprehensive understanding of this phenomenon. While SNRIs are generally recognized as a distinct category of antidepressants, they exhibit diverse pharmacological characteristics that contribute to their unique profiles within this class; the degree to which these variations will lead to notable clinical disparities remains uncertain.

## Figures and Tables

**Table 1:** The potential inhibitory and stimulatory effects of psychiatric drugs [11,14,21,24].

SSRI (Trade name)	1A2	2C9/10	2C19	2D6	3A3/4
Citalopram (Celexa)	−	−	−	++	−
Escitalopram (Lexapro)	−	−	−	++	−
Fluoxetine (Prozac)	−	++	++	+++	+
Fluvoxamine (Luvox)	+++	+++	+++	−	++
Sertraline (Zoloft)	−	−	−	−	
Paroxetine (Paxil)	−	−	−	−	−
SNRIs	1A2	2C9/10	2C19	2D6	3A3/4
Duloxetine (Cymbalta)	−	−	−	++	−
Venlafaxine (Effexor ER)	−	−	−	−	−
Newer Antidepressants	1A2	2C9/10	2C19	2D6	3A3/4
Bupropion (Wellbutrin)	??	??	??	+++	??
Nefazodone (Serzone)	−	−	−	−	+++

?? = unknown; + = mild effect (20–50%); ++ = moderate effect (50–150%); +++ = substantial effect (>150); − = no or minimal effect (<20).

## Data Availability

All data are available upon request.
